# A randomized, controlled, double-blind crossover study on the effects of isoeffective and isovolumetric intravenous crystalloid and gelatin on blood volume, and renal and cardiac hemodynamics

**DOI:** 10.1016/j.clnu.2019.09.011

**Published:** 2020-07

**Authors:** Christopher R. Bradley, Damian D. Bragg, Eleanor F. Cox, Ahmed M. El-Sharkawy, Charlotte E. Buchanan, Abeed H. Chowdhury, Ian A. Macdonald, Susan T. Francis, Dileep N. Lobo

**Affiliations:** a Gastrointestinal Surgery, Nottingham Digestive Diseases Centre and National Institute for Health Research (NIHR) Nottingham Biomedical Research Centre, Nottingham University Hospitals NHS Trust and University of Nottingham, Queen's Medical Centre, Nottingham, NG7 2UH, UK; b Sir Peter Mansfield Imaging Centre, University Park, University of Nottingham, NG7 2RD, UK; c School of Life Sciences, University of Nottingham, Queen's Medical Centre, Nottingham, NG7 2UH, UK; d MRC Versus Arthritis Centre for Musculoskeletal Ageing Research, University of Nottingham, Queen's Medical Centre, Nottingham, NG7 2UH, UK

**Keywords:** Balanced crystalloids, Balanced gelatin solution, Magnetic resonance imaging, Randomized controlled study, Cardiac output, Renal blood flow

## Abstract

**Background & aims:**

Blood volume expanding properties of colloids are superior to crystalloids. In addition to oncotic/osmotic properties, the electrolyte composition of infusions may have important effects on visceral perfusion, with infusions containing supraphysiological chloride causing hyperchloremic acidosis and decreased renal blood flow. In this non-inferiority study, a validated healthy human subject model was used to compare effects of colloid (4% succinylated gelatin) and crystalloid fluid regimens on blood volume, renal function, and cardiac output.

**Methods:**

Healthy male participants were given infusions over 60 min > 7 days apart in a randomized, crossover manner. Reference arm (A): 1.5 L of Sterofundin ISO, isoeffective arm (B): 0.5 L of 4% Gelaspan®, isovolumetric arm (C): 0.5 L of 4% Gelaspan® and 1 L of Sterofundin ISO (all B. Braun, Melsungen, Germany). Participants were studied over 240 min. Changes in blood volume were calculated from changes in weight and hematocrit. Renal volume, renal artery blood flow (RABF), renal cortex perfusion and diffusion, and cardiac index were measured with magnetic resonance imaging.

**Results:**

Ten of 12 males [mean (SE) age 23.9 (0.8) years] recruited, completed the study. Increase in body weight and extracellular fluid volume were significantly less after infusion B than infusions A and C, but changes in blood volume did not significantly differ between infusions. All infusions increased renal volume, with no significant differences between infusions. There was no significant difference in RABF across the infusion time course or between infusion types. Renal cortex perfusion decreased during the infusion (mean 18% decrease from baseline), with no significant difference between infusions. There was a trend for increased renal cortex diffusion (4.2% increase from baseline) for the crystalloid infusion. All infusions led to significant increases in cardiac index.

**Conclusions:**

A smaller volume of colloid (4% succinylated gelatin) was as effective as a larger volume of crystalloid at expanding blood volume, increasing cardiac output and changing renal function. Significantly less interstitial space expansion occurred with the colloid.

**Trial registration:**

The protocol was registered with the European Union Drug Regulating Authorities Clinical Trials Database (https://eudract.ema.europa.eu) (EudraCT No. 2013-003260-32).

## Abbreviations used

ADC: Apparent diffusion coefficient
ASL: Arterial spin labelling
β-NAG: N-acetyl-beta-glucosaminidase
BSA: Body surface area
CV: Coefficient of variance
DWI: Diffusion weighted imaging
ELISA: Enzyme-linked immunosorbent assay
GDFT: Goal-directed fluid therapy
IQR: Interquartile range
KIM-1: Kidney injury molecule 1
MR: Magnetic resonance
MRCP: Magnetic resonance cholangiopancreatography
MRI: Magnetic resonance imaging
NGAL: Neutrophil gelatinase-associated lipocalin
PC MRI: Phase contrast magnetic resonance imaging
RABF: Renal artery blood flow
RARE: Rapid acquisition with relaxation enhancement
SEM: Standard error of the mean
SID_a_: Apparent strong ion difference

## 1 Introduction

Restoration of blood volume in the perioperative period or in critically ill patients can be achieved with infusions of either crystalloids or colloids. However, it is clear that for a given volume of infusate, the blood volume expanding properties of colloids are superior to crystalloids. Work performed in healthy participants comparing the effects of 0.9% saline, 4% succinylated gelatin and 6% hydroxyethyl starch on blood volume expansion showed that colloids were three times as effective as crystalloids for this purpose [1]. Although the blood volume expanding efficacy of colloids is approximately 70% in patients undergoing laparoscopic cholecystectomy [2], in critically ill patients, volume for volume, colloids may be only up to 1.3 times as effective as crystalloids [3].

Colloids distribute predominantly in the intravascular compartment and this property may have important consequences for visceral blood supply and, thus, function. Crystalloids tend to distribute mainly in the interstitial fluid space, giving rise to tissue edema, and when given in excess, may have adverse effects on gastrointestinal function and wound healing [[4], [5], [6], [7]]. Colloid boluses have also been used intraoperatively to increase stroke volume (goal directed fluid therapy – GDFT), and, thereby, improve tissue perfusion and outcome [8]. A recent study has, however, demonstrated that crystalloids may be as effective as colloids for GDFT [9].

In addition to oncotic properties, the electrolyte composition of infusions may have important effects on visceral perfusion. Data derived from both healthy participant and patient studies have shown that infusions which contain a supraphysiological concentration of chloride can cause significant hyperchloremic acidosis [1,[10], [11], [12], [13], [14], [15], [16], [17]], and reduce renal arterial blood flow and perfusion [10].

The use of balanced crystalloids such as Hartmann's solution, Ringer's lactate, PlasmaLyte 148 (all Baxter Healthcare, Deerfield, IL, USA) and Sterofundin® ISO (B. Braun, Melsungen, Germany), with a sodium and chloride content closer to that of plasma, may achieve better acid-base balance and renal function, less tissue edema, nausea and vomiting, and possibly better survival [11]. Nevertheless, most of the older colloids are suspended in 0.9% saline and this is accompanied by the risk of hyperchloremic acidosis and its sequelae. It is only recently that colloids suspended in balanced solutions have become available.

Our group has previously used magnetic resonance imaging (MRI) to assess renal hemodynamics non-invasively during fluid delivery [10,18]. Phase contrast MRI (PC-MRI) is used to quantify renal artery blood flow (RABF), and Arterial Spin Labeling (ASL) is used to non-invasively map renal perfusion spatially without the need for contrast agents. We showed that when crystalloids were studied, the hyperchloremic acidosis caused by the infusion of 2 L 0.9% saline was associated with a decrease in renal artery flow velocity and renal cortical tissue perfusion when compared with the infusion of a balanced crystalloid [10]. However, in another study we demonstrated that while renal cortical tissue perfusion increased after infusion of 1 L 6% hydroxyethyl starch suspended in a balanced crystalloid, it did not change from baseline after the infusion of 6% hydroxyethyl starch suspended in 0.9% saline, suggesting that the effects of colloids are different from that of crystalloids [18].

Hence, in this non-inferiority study, we aimed to use a validated healthy male participant model previously developed by us [1,[10], [11], [12],^14^,^18^] to compare the effects of colloid (4% gelatin) and crystalloid regimens on RABF and renal cortex perfusion [10,18] as well as renal volume and renal cortex diffusion [apparent diffusion coefficient (ADC)] using diffusion weighted imaging (DWI). In addition, we assessed cardiac index.

The primary objective was to determine the differential impact of isoeffective [using different volumes of crystalloid (1.5 L) and colloid (0.5 L) with a similar plasma volume expanding capacity [1]] and isovolumetric infusions of crystalloid and colloid (1.5 L crystalloid *vs*. 1 L crystalloid + 0.5 L colloid) on blood volume expansion. Secondary endpoints included changes in weight, hematological and serum biochemical parameters, urinalysis, aortic blood flow and cardiac output, as well as renal volume, RABF, and renal cortex perfusion and diffusion.

## 2 Methods

This non-inferiority randomized, double-blind, crossover study was performed at a university teaching hospital. Twelve healthy males (aged 20–28 years, BMI 20–25 kg/m^2^) were recruited after obtaining written informed consent. Those with abnormal blood parameters on the day before each study, acute illness in the preceding 6 weeks, taking regular medication, a history of substance abuse, hypersensitivity to gelatin solutions or to any of the other infusion ingredients or having factors contraindicating MRI were excluded. It was decided to withdraw participants developing adverse events from the study. An adverse event was defined as any unfavorable and unintended sign, symptom, syndrome or illness that develops or worsens during the period of observation in the study. The UK National Research Ethics Service Committee East Midlands (Ref. 13/EM/0363) and the Medicines and Healthcare Products Regulatory Agency granted approvals. The protocol was registered with the European Union Drug Regulating Authorities Clinical Trials Database (https://eudract.ema.europa.eu) (EudraCT No. 2013-003260-32).

### 2.1 Baseline assessment

Participants reported 24 h prior to the study day to provide a blood and urine sample, and were asked to collect a 24-h urine sample for baseline creatinine clearance. Urine was analyzed for osmolality, neutrophil gelatinase-associated lipocalin (NGAL), N-acetyl-beta-glucosaminidase (β-NAG) and kidney injury molecule-1 (KIM-1), blood was analyzed for blood chemistry parameters to ensure that no concurrent electrolyte abnormalities were present. Height and weight was also recorded. Participants were instructed to abstain from alcohol, caffeine and nicotine for at least 24 h prior to each arm of the study. On the study day, participants presented to the MRI center at 8:00 am having fasted from the previous night. After providing a baseline urine sample, the baseline weight was recorded and a 16-G venous cannula inserted into each antecubital fossa. Blood sampling was performed after lying supine for at least 10 min for blood chemistry parameters including hemoglobin, hematocrit, serum electrolytes, urea, creatinine, albumin, and osmolality.

### 2.2 Infusions

All the study participants received the following infusions over 60 min on separate occasions at least 7 days apart and in a random order (Table 1).

A) 1.5 L of Sterofundin ISO – 217.5 mmol sodium and 190.5 mmol chloride (reference arm).
B) 0.5 L of 4% Gelaspan® – 75.5 mmol sodium and 51.5 mmol chloride (isoeffective arm).
C) 0.5 L of 4% Gelaspan® and 1 L of Sterofundin ISO – 220.5 mmol sodium and 178.5 mmol chloride (isovolumetric arm).

**Table 1:** Composition of infusions.

	4% Gelaspan®	Sterofundin ISO®
Colloid molecular weight	26 500 Da	
Sodium (mmol/L)	151	145
Chloride (mmol/L)	103	127
Potassium (mmol/L)	4	4
Calcium (mmol/L)	1	2.5
Magnesium (mmol/L)	1	1
Acetate (mmol/L)	24	24
Malate (mmol/L)		5
Strong ion difference (mmol/L)	52	22
Sodium supplied as	NaCl, 5.55 g/L	NaCl, 6.8 g/L
pH	7.4	5.1–5.9
Theoretical osmolarity, mOsm/L	284	309

Both infusions manufactured by B. Braun, Melsungen, Germany.

All infusions were manufactured by B. Braun, Melsungen, Germany.

### 2.3 Measurements and end points

Blood was sampled at 0 (baseline), 30, 60, 90, 120, 180 and 240 min after commencing the infusion. Subjects voided their bladder on arrival at the MRI center at 8:00 am. After commencement of the infusions they were allowed to void urine at will and had to void again at 240 min, with all of the urine voided being collected. An aliquot of the urine pooled over the 4-h period was analyzed for osmolality and concentrations of urea, creatinine, sodium, potassium, chloride, total proteins, microalbuminuria and urinary NGAL, β-NAG and KIM-1. A further 24-h urine collection was performed from 8:00 am on the study day until 24 h post infusion on the following day. The urine collected over the 240 min of the study was then pooled with urine collected from then until the first voided sample on the next morning to constitute a 24-h urine collection. Urine pooled over the 24-h period was analyzed for the previously mentioned parameters. Weight was recorded at baseline, 100, 120, 180 and 240 min.

### 2.4 Hematological and biochemical blood analyses

All analyses were performed according to standard methods [1,[10], [11], [12],^14^,^18^]. Assays for NGAL (Human Lipocalin-2/NGAL Quantikine ELISA Kit, R&D Systems Inc., Minneapolis, USA), KIM-1 (Human TIM-1/KIM-1/HAVCR Quantikine ELISA Kit, R&D Systems Inc., Minneapolis, USA) and β-NAG (NAG test kit, PPR Diagnostics Ltd., London, UK) were performed using ELISA in accordance with the instructions provided by the manufacturers.

### 2.5 Derived values

Blood volume at time 0 was estimated according to the method described by Nadler et al. [19] and changes in blood volume after the infusions were calculated from changes in hematocrit, using formulae we have described previously [1,2,10,18]. The apparent strong ion difference (SID_a_) was calculated as described by Stewart (SID_a_ = [Na^+^]+[K^+^]-[Cl^−^]) [20]. Change in interstitial fluid volume was calculated by subtracting change in blood volume from change in weight.

### 2.6 MRI protocol

Cardiac and renal MRI measurements were collected on a 3.0 T Philips Achieva MR scanner (Philips Healthcare Systems, Best, Netherlands). The MRI protocol consisted of a series of non-invasive MR measurements to assess cardiac and renal function.

Cardiac MRI data were collected using phase contrast (PC)-MRI to assess cardiac output. Data were then corrected for body surface area (BSA) to compute cardiac index (CI). Renal MRI data were collected using structural images to assess total renal volume, PC-MRI was acquired to determine left RABF, Arterial Spin Labelling (ASL) was used to determine renal cortex tissue perfusion incorporating an estimation of renal T_1_ in the quantification of perfusion [21]. Diffusion Weighted Imaging (DWI) was used to determine renal cortex apparent diffusion coefficient (ADC).

**Fig. 1:**
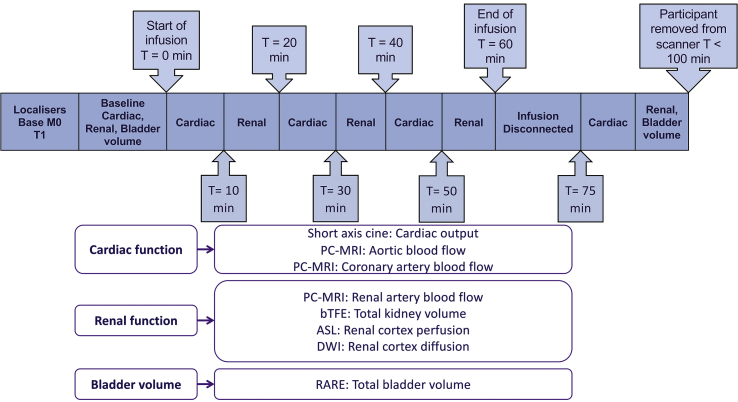
Diagram showing the protocol for MRI scan collection in each of the infusion sessions to assess cardiac and renal function. The protocol comprised scans to be collected at baseline, repeated at 20-min intervals over the 60 min infusion, and repeated once post-infusion to assess the time-course of the renal and cardiac response. Note, acquired timings may deviate slightly from this protocol timing due to blood sampling and infusion technicalities, timings provided in Figures in the Results section show the actual scan acquisition time (mean time across participants).

Each series of cardiac and renal images were collected in 10 min, with the cardiac and renal scans being interleaved throughout the infusion. In total there were three repeats of the cardiac and three renal scans collected during the infusion, and a further repeat of each post-infusion to assess the time-course of the response (Fig. 1).

Bladder volume was measured using MR cholangiopancreatography (MRCP) with a Rapid Acquisition with Relaxation Enhancement (RARE) sequence at baseline and <40 min after the end of the infusion. Details of the MRI scanning protocols and analysis methods are described in the Supplementary Methods.

### 2.7 Sample size

This was a pilot study and we assumed that the blood volume expanding effects of the three fluid regimens would be equivalent (non-inferiority). Based on our previous work [10,18], we determined that a sample size of 10 would be adequate to obtain meaningful data for a pilot study. Assuming a drop-out rate of 15%, we sought to recruit 12 participants.

### 2.8 Randomization, allocation concealment and blinding

A randomization sequence was created using http://www.randomization.com by the Nottingham Clinical Trials Pharmacy. Randomization was performed using sequentially numbered paired sealed opaque envelopes. A nurse not involved in the study was responsible for blinding the investigational products. The infusion bags were covered with opaque bags and the data shown on the screen of the automated infusion pump was masked from the investigators. The randomization code was broken after completion of data analysis.

### 2.9 Statistical analysis

Statistical analysis was performed using GraphPad Prism (v 7.03) for Windows statistical software package (GraphPad Software Inc., La Jolla, CA). A Shapiro–Wilk test was used to test the normality of the data. Normally distributed group data quantitative variables were expressed as mean [standard error of mean (SEM)], and skewed data as median and interquartile range. A two-way ANOVA with Bonferroni correction was used for comparing two groups with multiple time point comparisons. A one-way ANOVA with Kruskal–Wallis test was used for single comparisons across all three groups. Comparisons between two groups for non-parametric data were analyzed using a Wilcoxon signed-rank test. A student paired t-test was used for comparing two groups where the distribution was normal. The differences were considered statistically significant at P < 0.05.

## 3 Results

Twelve participants, with a mean (SEM) age of 23.9 (0.8) years were recruited to the study over a 13-month period. Two participants were withdrawn from the study due to adverse events. One participant developed persistent tachycardia up to 115 beats per minute during one of the infusions (0.5 L colloid), this settled shortly after terminating the infusion with no further adverse effects. One participant was excluded from analysis due to excessive sweating during two of the infusion sessions and was biochemically more dehydrated following infusion, he required no medical attention. Analysis was performed on data obtained from the remaining 10 participants. Baseline clinical and MRI parameters prior to each infusion arm, and the coefficient of variation in MRI measures are summarized in Table 2.

**Table 2:** Baseline clinical and MRI parameters prior to each infusion.

	Before infusion A (1.5 L crystalloid) n = 10	Before infusion B (0.5 L colloid) n = 10	Before infusion C (1 L crystalloid + 0.5 L colloid) n = 10	Coefficient of Variation of baseline MR measures across treatment
**Clinical Parameters**				
Weight (kg)	74.5 (1.8)	74.1 (1.6)	73.9 (1.6)	
Height (m)	1.81 (0.02)	1.81 (0.02)	1.81 (0.02)	
Body mass index (kg/m^2^)	22.7 (0.5)	22.6 (0.5)	22.5 (0.4)	
Hemoglobin (g/L)	150.1 (2.4)	147.8 (2.9)	147.2 (3.0)	
Hematocrit (L/L)	0.439 (0.006)	0.432 (0.007)	0.431 (0.007)	
Serum chloride (mmol/L)	102.9 (0.4)	103.6 (0.6)	103.8 (0.8)	
Serum apparent strong ion difference (mmol/L)	41.2 (0.8)	39.9 (0.6)	40.5 (0.5)	
Serum bicarbonate (mmol/L)	28.2 (0.7)	27.5 (0.5)	27.3 (0.6)	
Serum albumin (g/L)	42.4 (0.8)	41.7 (1.0)	42.8 (0.5)	
Serum osmolality (mOsm/kg)	291 (0.8)	291 (1.0)	291 (0.9)	
Serum creatinine (*μ*mol/L)	78.6 (2.7)	77.6 (2.6)	76.4 (3.2)	
Creatinine clearance (ml/min)	139.6 (13.0)	131.8 (18.6)	127.3 (8.2)	
Calculated blood volume (L)	5.2 (0.1)	5.2 (0.1)	5.2 (0.1)	
**MRI Parameters**				
Aortic flux (ml/s)	91 (4)	91 (4)	93 (4)	8.2 (2.8)
Cardiac index from aortic flow (L/min/m^2^)	2.82 (0.1)	2.83 (0.1)	2.89 (0.1)	8.2 (3.6)
Cardiac index from short axis cine (L/min/m^2^)	1.55 (0.1)	1.55 (0.1)	1.52 (0.1)	16 (9)
Renal artery blood flow velocity (cm/s)	6.3 (0.6)	8.0 (0.6)	7.0 (0.4)	12 (5)
Renal cortical tissue perfusion (ml/100 g/min)	215 (14)	235 (17)	235 (9)	9.1 (4.4)
Left global renal perfusion (ml/100 g/min)	215 (20)	262 (14)	231 (13)	12 (5)
Total kidney volume (ml)	351 (25)	365 (23)	359 (24)	4.2 (2.8)
Cortex T_1_ (ms)	1026 (9)	1021 (9)	1020 (11)	2.3 (1.1)
Medulla T_1_ (ms)	1340 (12)	1327 (13)	1335 (21)	2.9 (2.4)
Bladder volume (ml)	37 (9)	51 (23)	51 (12)	40 (32)

All values mean (SEM). There were no statistically significant differences (p > 0.05) between the groups when baseline parameters were compared.

### 3.1 Changes in weight, hematocrit, hemoglobin, blood volume, interstitial fluid volume and biochemical parameters

**Fig. 2:**
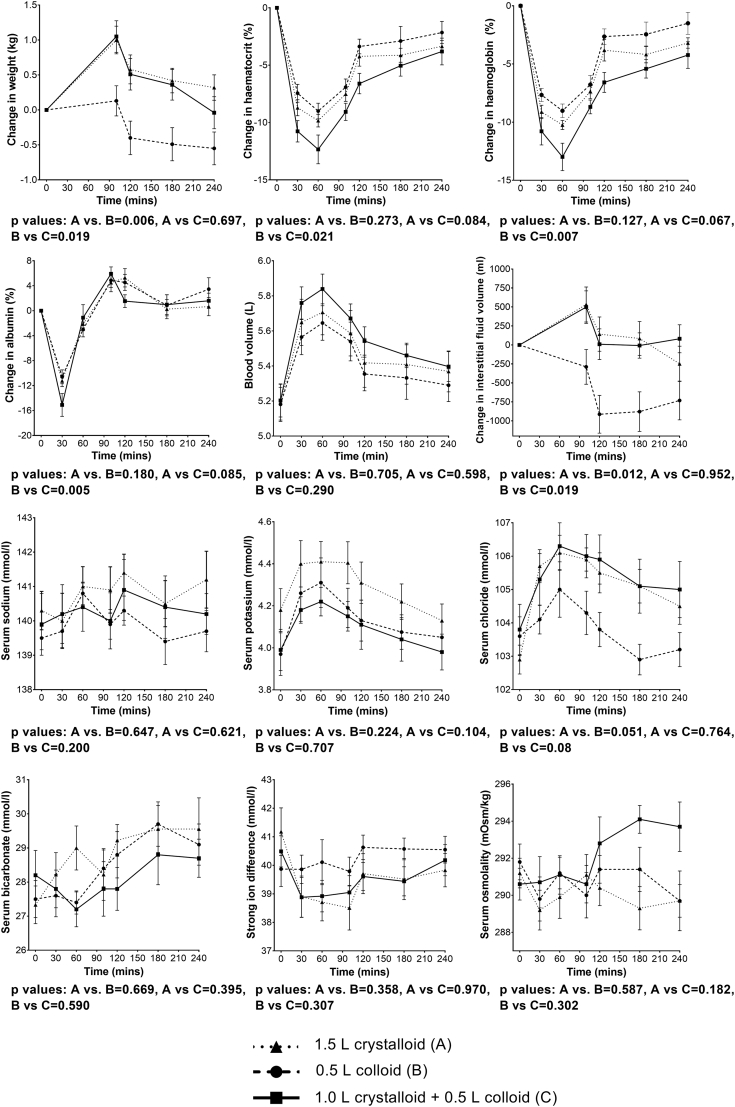
Changes in body weight, hematocrit, hemoglobin, calculated blood volume and interstitial fluid volume, as well as serum biochemistry. Note weight was recorded at baseline and post MRI scanning.

Changes in body weight, hematocrit, hemoglobin, calculated blood volume and interstitial fluid volume, as well as serum biochemistry are shown in Fig. 2. As expected, increase in body weight and extracellular fluid volume were significantly less after the colloid infusion (B) when compared with the crystalloid (A) and crystalloid + colloid infusions (C). For most other parameters, there was no statistically significant difference when the groups were compared. Changes in urinary parameters are summarized in Table 3.

**Table 3:** Urinary responses.

	Infusion A (1.5 L crystalloid)	Infusion B (0.5 L colloid)	Infusion C (1.0 L crystalloid + 0.5 L colloid)	*p* (A *vs.* B)	*p* (A *vs.* C)	*p* (B *vs.* C)
Time to first micturition after start of infusion (min)	106 (4)	106 (5)	123 (14)	0.89	0.27	0.17
Postinfusion urinary volume at 240 min (ml)	621 (99)	517 (68)	826 (97)	0.20	0.13	**<0.05**
Preinfusion urinary osmolality (mOsm/kg)	689 (48)	634 (76)	712 (56)	0.21	0.79	0.28
Postinfusion urinary osmolality at 240 min volume (mOsm/kg)	466 (56)	473 (60)	378 (30)	0.88	0.26	0.19
Total postinfusion urinary sodium over 24 h (mmol)	242 (21)	157 (27)	186 (28)	**0.02**	0.06	0.19
Total postinfusion urinary sodium at 240 min (mmol)	69 (12)	49 (7)	72 (10)	0.12	0.87	**<0.05**
Urine sodium at 240 min as percentage of sodium infused	32 (6)	64 (10)	33 (5)	**<0.05**	0.92	**<0.05**
Total postinfusion urinary potassium over 24 h (mmol)	86 (9)	69 (12)	63 (9)	0.09	0.07	0.89
Total postinfusion urinary chloride at 240 min (mmol)	78 (13)	63 (12)	75 (11)	0.34	0.87	0.07
Urine chloride at 240 min as percentage of chloride infused	41 (7)	122 (24)	42 (6)	**<0.05**	0.86	**<0.05**

n = 10, all values mean (SEM). Student paired t test. p values in bold indicate statistically significant differences.

### 3.2 Urinary NGAL, KIM-1, β-NAG

Although some differences were noted in urinary NGAL, KIM-1 and β-NAG at the measured time points, all values remained in the normal range (data not shown).

### 3.3 Changes in renal volume, renal artery blood flow, renal global and cortical tissue perfusion, and renal cortex diffusion determined by MRIs

**Fig. 3:**
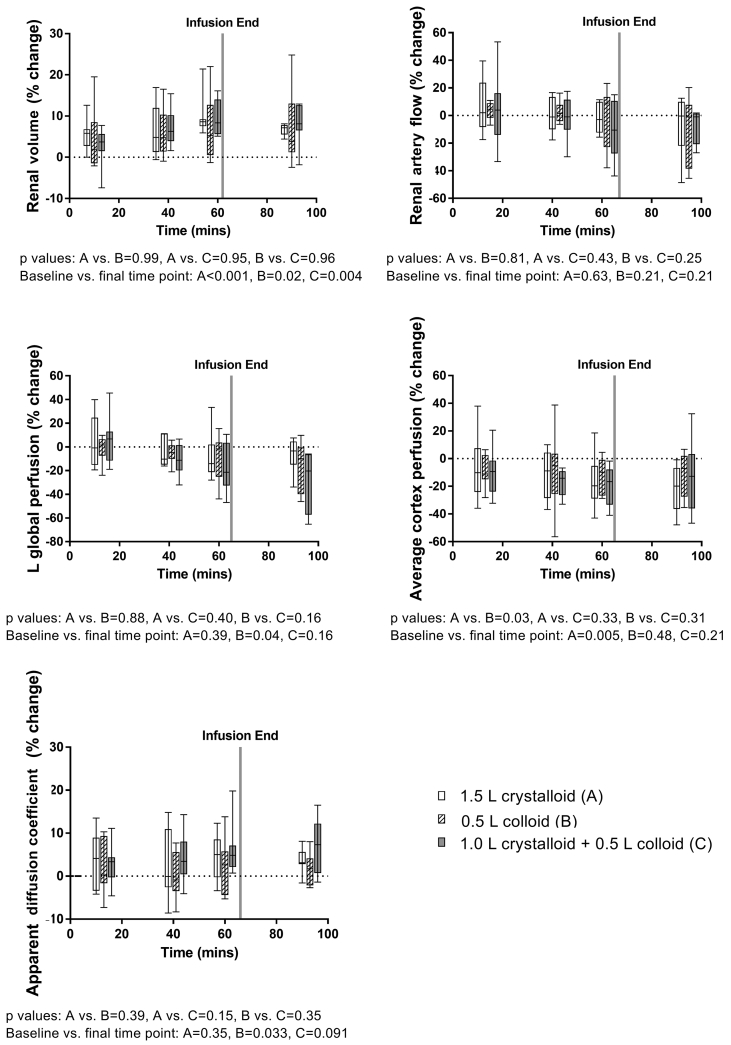
Percentage change in renal MRI parameters (renal volume, renal artery flow, left renal global perfusion, average cortex perfusion and apparent diffusion coefficient) during each infusion.

The percentage change in renal MRI parameters, as measured from baseline are shown in Fig. 3. All infusions increased renal volume, this increase was statistically significant at 90 min when compared with baseline for each infusion [A (P < 0.001), B (P = 0.02), C (P = 0.004)]. However, there were no statistically significant differences between the infusions. There was no significant difference in RABF from baseline for any time point across the infusions or any difference between infusions. However, when left RABF was corrected for renal volume change yielding a measure of global perfusion of the left kidney at each time point, a significant decrease was observed between baseline and 95 min after the start of the 0.5 L colloid infusion (P = 0.037). No significant change in global perfusion of the left kidney was observed for the crystalloid or combined crystalloid and colloid infusion. Renal cortex perfusion across both kidneys, as determined by ASL, decreased from baseline to 93 min after the crystalloid infusion [with a 23% decrease from baseline (P = 0.005)] and the colloid infusion [with a 14% decrease from baseline (P = 0.048)]. No significant difference was found in renal cortex perfusion for the combined crystalloid and colloid infusion, or between infusions. There was a significant increase in renal cortex ADC from baseline to 95 min after the crystalloid infusion [4.2% increase from baseline (P = 0.033)], and a trend for an increase in the combined crystalloid and colloid infusion (P = 0.09), whilst no significant change was observed in the colloid infusion.

### 3.4 Changes in heart rate, stroke volume, aortic flow and cardiac index

**Fig. 4:**
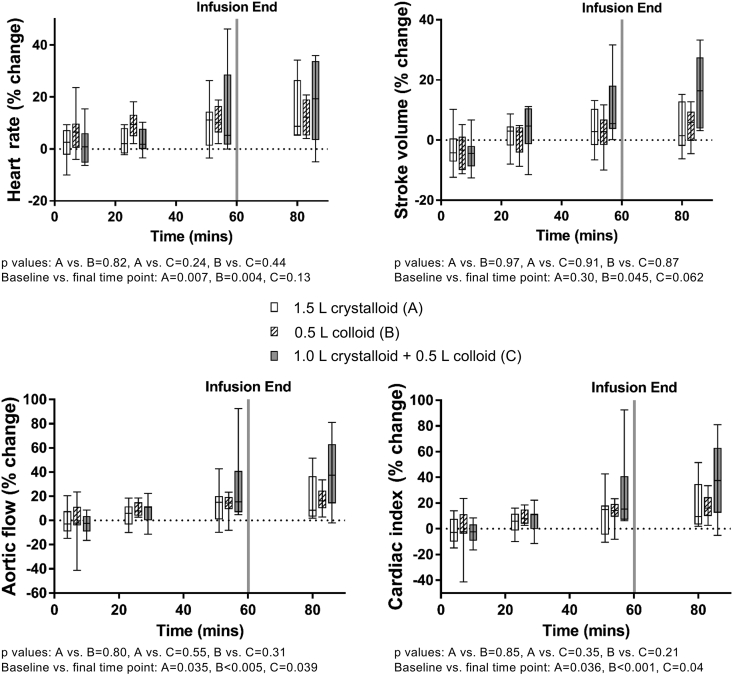
Percentage change in cardiac MRI parameters (heart rate, stroke volume, aortic flow and cardiac index) during each infusion.

Changes in cardiac MRI measures are shown in Fig. 4. All infusions led to a significant increase at 83 min after baseline in aortic flow [crystalloid (P = 0.035), colloid (P < 0.005), combined crystalloid and colloid (P = 0.039)], and a significant increase in cardiac index [crystalloid (P = 0.036), colloid (P < 0.001), combined crystalloid and colloid (P = 0.04)]. Aortic stroke volume significantly increased for the colloid infusion (P = 0.045) with a trend for increase in the combined crystalloid and colloid infusion (P = 0.062), no significance was observed in the crystalloid infusion. Heart rate significantly increased at 83 min for the crystalloid infusion (P = 0.007) and the colloid infusion (P = 0.004), no significant difference was observed for the combined crystalloid and colloid infusion.

## 4 Discussion

### 4.1 Main findings

This study has confirmed our previous work, showing that the blood volume expanding potential of 0.5 L of colloid (gelatin) is similar to that of 1.5 L of crystalloid in healthy participants [1]. In addition, it has shown that the blood volume expansion produced by a combination of 1 L of crystalloid with 0.5 L of colloid was not statistically different from that produced by separate infusions of either 1.5 L of crystalloid or 0.5 L of colloid. Significant changes in response to the infusions were seen at final measurement compared to baseline with an increase in renal volume, a decrease in global renal perfusion as measured by PC-MRI corrected for renal volume and renal cortex perfusion as assessed by ASL. Perfusion measured using ASL was deemed to be the more robust method of renal hemodynamics, providing a smaller CV than estimating global perfusion by correcting renal artery blood flow by kidney volume. This finding has also been shown in a previous study [21]. The significant increase in renal cortex ADC following the crystalloid infusion could be explained by the increase in the amount of water in the interstitial space of the kidney with crystalloids, which could be a result of decreased glomerular filtration and is consistent with the change in renal volume for the crystalloid infusion [22].

The changes in renal hemodynamics were, however, of a smaller magnitude when compared with our previous work where we induced mean serum chloride concentrations in excess of 108 mmol/L [10]. In addition, markers of acute kidney injury remained in the normal range. All three infusions produced >10% time-related increase in heart rate, stroke volume and cardiac index, but there was no statistically significant difference between the effects of the three infusions.

### 4.2 Results in context of published literature

The results of this study have also further validated the reproducibility of the model we have developed to study responses of healthy participants to intravenous fluid infusions [1,[10], [11], [12],^14^,^18^]. As shown previously [1,[10], [11], [12],^14^,^18^], all the infusion regimens used in the present study produced a dilutional effect on hematocrit, hemoglobin and serum albumin concentration that was reversed as the fluid was excreted in the urine. The crystalloid (1.5 L) and crystalloid + colloid (1 L + 0.5 L respectively) infusions expanded the calculated interstitial fluid space to the same extent, indicating the propensity to produce edema. In contrast, the colloid infusion produced a contraction of the interstitial space, suggesting that the increased colloid oncotic pressure helps draw fluid from the interstitial into the intravascular space. Urine output during the study period was very similar after the infusion of 1.5 L of crystalloid and 0.5 L of colloid, but was slightly greater after the combined crystalloid and colloid infusion.

Compared with infusions of 0.9% saline [1,10,14], all infusions used in this study produced only a small elevation in serum chloride concentration and a 2 mmol/L fall in the strong ion difference, reflecting the reduced chloride concentration of the infusions when compared with 0.9% saline. The changes in serum bicarbonate and potassium concentrations and serum osmolality were also relatively small and for all these measures there were no statistically significant differences between the three infusions. In our previous study [10], we showed that the elevation of serum chloride concentration to >108 mmol/L after the infusion of 2 L 0.9% saline led to a significant fall in both renal artery flow velocity and renal cortical tissue perfusion. However, in the present study, the chloride content of all the infusions was less than that of 0.9% saline and the maximum that the serum chloride concentration reached was around 106 mmol/L (Fig. 2). In addition, there was no significant difference in the fall in strong ion difference between the three infusions. This strengthens the hypothesis that it is the hyperchloremic (≥108 mmol/L) acidosis produced by large chloride loads that has an adverse effect on renal hemodynamics [10,11,23,24]. A hypothesis for the mechanisms of hyperchloremia on reducing renal blood flow and glomerular filtration rate, and ultimately decreasing urinary output and sodium excretion has been proposed [11]. This involves chloride from the high chloride concentration filtrate in the distal tubule of the nephron crossing the basement membrane and causing depolarization of the macula densa and release of adenosine, which acts on the A_1_ receptors of the renal vasculature causing vasoconstriction. This phenomenon probably does not occur at a chloride concentration of around 106 mmol/L, and may, thereby, explain the results of our study. Although there was an increase in renal volume by about 5% after all three infusions, indicating some renal edema, there was probably not a high enough increase in intraorgan tissue pressure to disrupt RABF significantly. Interestingly, we have also demonstrated that the infusion of colloid in the form of 4% succinylated gelatin had no adverse effect on renal hemodynamics or markers of kidney injury such as NGAL, β-NAG and KIM-1.

There was a steady increase in heart rate and cardiac index after the three infusions. There was a >10% increase in these parameters when the values at the end of the infusions were compared with baseline. This suggests that even in relatively “euvolemic” healthy participants, infusions of colloids and crystalloids increase stroke volume and cardiac index. To some extent, this response in healthy euvolemic participants may explain why, in some studies using “flow directed fluid therapy”, patients received excessive amounts of fluids intraoperatively [9]. Moreover, as the differences seen after crystalloids and colloids were similar, it is possible that crystalloids may have the same effect as colloids when used for “flow-directed fluid therapy” [9]. However, crystalloids are more likely to cause interstitial edema than colloids.

We have demonstrated that infusions containing higher amounts of sodium and chloride lead to a disproportionately higher retention of sodium (Table 3). This effect is believed to be due to an increase in serum chloride concentrations [10,11].

Renal perfusion decreased across all arms of the study. These changes are believed to be a reflection of the action of hyperchloremia leading to a decrease in RABF and an increase in renal volume. This, however, merits some clarification in light of previous work [10,18]. The hyperchloremic acidosis caused by the infusion of 2 L 0.9% saline was associated with a decrease in renal artery flow velocity and renal cortical tissue perfusion when compared with the infusion of a balanced crystalloid [10]. Using 1 L infusions of colloids, we have previously demonstrated that while renal cortical tissue perfusion increased after infusion of 6% hydroxyethyl starch suspended in a balanced crystalloid, it did not change from baseline after the infusion of 6% hydroxyethyl starch suspended in 0.9% saline, suggesting that the effects of colloids are different from that of crystalloids [18]. More importantly, in the previous studies [10,18], the peak serum chloride concentrations were higher (>108 mmol/L) after 0.9% saline and hydroxyethyl starch suspended in 0.9% saline than after any of the infusions studied in the present study.

### 4.3 Strengths of the study

This experiment has used a well validated model and state of the art MRI techniques that have been shown to have low coefficients of variance (<10%) [21] (Table 2) to study the effects of intravenous fluid infusions on serum and urinary biochemistry and renal and cardiac hemodynamics in healthy human participants. The age and body weight of the participants were within a narrow range, ensuring homogeneity. The fact that the baseline parameters were very similar prior to each infusion (Table 2), indicates that the participants were studied under similar conditions. Coefficients of Variation on MR parameters over 3 weeks assessed were lower than 10% for most MR measures including aortic flow; total kidney volume; renal cortex perfusion estimated with ASL; and apparent diffusion coefficient estimated with DWI.

### 4.4 Limitations of the study

This study has a few limitations. Healthy participants who were not hypovolemic were studied, and although the study yields valuable data in this group, the results may be different in patients who are hypovolemic or critically ill. Although state of the art MRI techniques were used, the nature of the protocol resulted in cardiac and renal measurements being made at different time points. Although there was little variability in the blood and urinary measurements, there was some variability in the MRI results. We would advise that future studies are conducted to also gather MR data at later time points post-infusion in order to observe measures as they return to a baseline state. In order to gather the five data points across the infusion, the participants in this cohort were supine and on the scanner bed for approaching 3 h, we would advise that future studies collect a similar amount of time points spread over a longer period of time in order to allow for bladder voiding, this would also allow the study of larger infusions similar to the amounts patients actually receive perioperatively.

## 5 Conclusion

MRI provides quantitative measures that are sensitive enough to observe dynamic changes in cardiac function as well as renal structure and function in response to intravenous infusions. A smaller volume of colloid (0.5 L) was as effective as a larger volume of crystalloid (1.5 L) at expanding the blood volume and increasing cardiac output. Significantly less expansion of the interstitial space was associated with an isoeffective volume of colloid. A significant increase in renal volume was associated with all infusions, renal cortex ADC increased significantly only for the crystalloid infusion which showed the most significant renal volume increase. A decrease in global renal cortex perfusion renal cortex perfusion as assessed by ASL was shown for both crystalloid and colloids, with the former driven solely by renal volume change and the later likely by both a reduction in RABF and increase in renal volume. Non-inferiority of the treatment with colloid (4% succinylated gelatin) was also confirmed. The results also indicate that a serum chloride concentration ≥108 mmol/L may be necessary to demonstrate adverse effects of hyperchloremia.

## Author contributions

CRB – study design, literature search, data collection, data analysis, data interpretation, writing of the manuscript and final approval.

DDB – study design, literature search, data collection, data analysis, data interpretation, writing of the manuscript and final approval.

EFC – study design, literature search, data collection, data analysis, data interpretation, writing of the manuscript and final approval.

AME-S – study design, literature search, data interpretation, writing of the manuscript and final approval.

CEB – study design, literature search, data collection, writing of the manuscript and final approval.

AHC – study design, literature search, data interpretation, writing of the manuscript and final approval.

IAM – study design, data interpretation, writing of the manuscript, critical review, and final approval.

STF – study design, literature search, data interpretation, writing of the manuscript, critical review, supervision and final approval.

DNL – study design, literature search, data interpretation, writing of the manuscript, critical review, supervision and final approval.

## Funding

This study was supported by the 10.13039/501100000265Medical Research Council [grant number MR/K00414X/1]; and 10.13039/501100000341Arthritis Research UK [grant number 19891]. This study was an investigator-initiated project funded by an unrestricted grant from 10.13039/100007534B. Braun, Melsungen, Germany. Support was also received from the 10.13039/501100000272National Institute for Health Research Nottingham Digestive Diseases Biomedical Research Unit.

## Role of funding bodies

The funders had no role in the study design, conduct of the study, data collection or analysis or interpretation, and writing of the paper or the decision to submit for publication. No payment has been received from any other source or agency. The corresponding author has full access to all the data in the study and has final responsibility for the decision to submit for publication.

This paper presents independent research funded by the National Institute for Health Research (NIHR). The views expressed are those of the authors and not necessarily the views of the NHS, the NIHR or the Department of Health.

## Conflict of Interest

CRB and DDB received travel grants from B. Braun for presentation of the data. IAM has received research funding from 10.13039/100007246Mars Inc. and serves on the advisory board of IKEA for unrelated work. DNL has received unrestricted research funding and speaker's honoraria from Fresenius Kabi, B. Braun, Shire and Baxter Healthcare. None of the other authors have any conflicts of interest to declare.
