# Supplementary material for: A randomized, controlled, double-blind crossover study on the effects of isoeffective and isovolumetric intravenous crystalloid and gelatin on blood volume, and renal and cardiac hemodynamics
Source: Clin Nutr. 2020 Jul;39(7):2070–9. doi: 10.1016/j.clnu.2019.09.011 (PMC7359406; doi:10.1016/j.clnu.2019.09.011)
Supplement: Multimedia component 1 [file mmc1.docx]

**Supplementary methods**

A series of scout images were initially acquired in three orthogonal planes to locate the kidneys, heart and vessels of interest to aid subsequent slice positioning. Baseline cardiac and renal MRI data were then collected comprising structural images to assess kidney volume, phase contrast (PC)-MRI to determine cardiac output and renal artery bulk flow, Arterial Spin Labelling (ASL) data to determine renal cortex tissue perfusion, and Diffusion Weighted Imaging (DWI) to determine the renal cortex apparent diffusion coefficient (ADC). These scans were repeated at 20 minute intervals over the course of the 60 min infusion and at 20 minutes post-infusion to assess the time-course of the response. Bladder volume was measured using MR cholangiopancreatography (MRCP) before and approximately 20 minutes after infusion end.

#### Cardiac output

PC-MRI was collected using an imaging plane placed perpendicular to the ascending aorta. Acquisition parameters comprised a single slice TFE sequence with 30 phases collected across the cardiac cycle with TE/TR 2.4/3.8ms, FA 15^o^, number of excitations 3, reconstructed resolution 1.05 × 1.05 × 10 mm^3^, velocity encoding 200 cm/s. The TFE factor depended on the subjects’ heart rate, data was acquired free breathing in approximately 1 minute. Analysis was performed using ViewForum software (Philips Medical Systems, Best, Netherlands). Arterial flow velocity (cm/s), area (mm^2^) and, hence flow (ml/s) were calculated over the cardiac cycle. Cardiac output (L/min) was computed by multiplying stroke volume (i.e. the area under the ﬂow curve within one cardiac cycle) and heart rate. Data were then body surface area (BSA) corrected to compute cardiac index (CI).

#### Renal volume

Coronal multi-slice balanced-Turbo-Field-Echo (bTFE) images were used to estimate renal volume, these comprised 30 slices of 1.75 x 1.75 x 7 mm^3^, with data collected in single breath hold. Analyze9® software (Mayo Clinic) was used to draw a region of interest (ROI) to determine left and right kidney volume within each bTFE image slice. Total renal volume was then calculated from the sum of the volume measures computed across all the slices across both kidneys.

#### Renal artery blood flow (RABF) and global perfusion

PC-MRI was performed using a single slice TFE technique with the imaging plane placed perpendicular to the left renal artery. Imaging parameters were: echo time (TE)/repetition time (TR) 3.4/7.5 ms, FA 25^o^, number of excitations 2, reconstructed resolution 1.17 × 1.17 × 6 mm^3^, velocity encoding 100 cm/s, with 20 phases collected across the cardiac cycle. Each renal artery measurement was acquired during a single 15-20 s breath hold. Analysis was performed using ViewForum software to compute mean arterial flow velocity (cm/s), mean area (mm^2^) and thus mean flux (ml/s) over the cardiac cycle for the left renal artery. Global perfusion of the left kidney was then calculated by correcting left renal artery blood flow by left kidney volume.

#### Renal cortex perfusion

Respiratory-triggered Arterial Spin Labelling (ASL) data (288 × 300 mm^2^ field of view (FOV), 3 × 3 × 8 mm^3^ voxel size) were collected using a balanced fast field echo (bFFE) readout scheme [TE/TR 2.1/4.1 ms, SENSE 2, flip angle (FA) 60^o^, low-high acquisition, and half-Fourier acquisition] with 25 ASL pairs collected in approximately 5 minutes. A base M_0_ equilibrium scan and T_1_ longitudinal relaxation time map (collecting 13 inversion times of 200, 300, 400, 500, 600, 700, 800, 900, 1000, 1100, 1200, 1300 and 1500 ms using a modified respiratory triggering scheme in a total scan time of less than 3 minutes [1] were acquired for quantification of renal tissue perfusion [2]. Individual perfusion weighted difference images (control-label pairs) were computed, inspected for motion (exclude >1 voxel movement) and realigned, and averaged to create a single perfusion-weighted (PWI) map. Mean renal cortical perfusion was calculated across both kidneys from the PWI maps, T_1_ maps and M_0_ scans in a kinetic model to calculate tissue perfusion (f) maps (in ml/100 g tissue/min) [1].

#### Renal cortex diffusion

To obtain specific information on water diffusion in the kidney, diffusion weighted images were acquired with a spin-echo–echo planar imaging (SE-EPI) sequence. Imaging acquisition parameters were a 288 × 288 mm^2^ FOV, 3 × 3 × 8 mm^3^ voxel size, and TE of 56 ms. Eleven diffusion weighting factors (b-values) were acquired of 0, 5, 20, 60, 120, 190, 270, 370, 470, 580, 700 s/mm^2^ to allow estimation of the apparent diffusion coefficient (ADC) and pure tissue molecular diffusion coefficient (D). To compute ADC maps, data was fit to the log of the exponential signal [1].

#### Bladder volume assessment

Bladder volume was measured using a single shot, fast spin echo sequence (similar to that used for MR cholangiopancreatography- MRCP, effective TE 283 ms) with 30 axial contiguous slices (reconstructed resolution 2 × 2 x 7 mm^3^) acquired free breathing. Volumes were assessed by manually tracing ROIs around the bladder on each slice using Analyze9® software (Mayo Clinic) and summing across the slices.

**References**

Cox EF, Buchanan CE, Bradley CR, et al. Multiparametric renal magnetic resonance Imaging: validation, interventions, and alterations in chronic kidney disease. Front Physiol 2017;8:696.

Gardener AG, Francis ST. Multislice perfusion of the kidneys using parallel imaging: image acquisition and analysis strategies. Magn Reson Med 2010; 63: 1627-1636.
